# Will Attention by Vaccine Developers to the Host’s Nuclear Hormone Levels and Immunocompetence Improve Vaccine Success?

**DOI:** 10.3390/vaccines7010026

**Published:** 2019-02-27

**Authors:** Robert E. Sealy, Bart G. Jones, Sherri L. Surman, Rhiannon R. Penkert, Stephane Pelletier, Geoff Neale, Julia L. Hurwitz

**Affiliations:** 1 Department of Infectious Diseases, St. Jude Children’s Research Hospital, Memphis, TN 38105, USA; bob.sealy@stjude.org (R.E.S.); bart.jones@stjude.org (B.G.J.); sherri.surman@stjude.org (S.L.S.); rhiannon.penkert@stjude.org (R.R.P.); 2 Department of Immunology, St. Jude Children’s Research Hospital, Memphis, TN 38105, USA; stephane.pelletier@stjude.org; 3 The Hartwell Center for Bioinformatics & Biotechnology, St. Jude Children’s Research Hospital, Memphis, TN 38105, USA; geoffrey.neale@stjude.org; 4 Department of Microbiology, Immunology and Biochemistry, University of Tennessee Health Science Center, Memphis, TN 38163, USA

**Keywords:** estrogen, vitamin A, vitamin D, nuclear hormone, nuclear hormone receptors, response elements, immunoglobulin heavy chain locus, antibody isotypes

## Abstract

Despite extraordinary advances in fields of immunology and infectious diseases, vaccine development remains a challenge. The development of a respiratory syncytial virus vaccine, for example, has spanned more than 50 years of research with studies of more than 100 vaccine candidates. Dozens of attractive vaccine products have entered clinical trials, but none have completed the path to licensing. Human immunodeficiency virus vaccine development has proven equally difficult, as there is no licensed product after more than 30 years of pre-clinical and clinical research. Here, we examine vaccine development with attention to the host. We discuss how nuclear hormones, including vitamins and sex hormones, can influence responses to vaccines. We show how nuclear hormones interact with regulatory elements of immunoglobulin gene loci and how the deletion of estrogen response elements from gene enhancers will alter patterns of antibody isotype expression. Based on these findings, and findings that nuclear hormone levels are often insufficient or deficient among individuals in both developed and developing countries, we suggest that failed vaccine studies may in some cases reflect weaknesses of the host rather than the product. We encourage analyses of nuclear hormone levels and immunocompetence among study participants in clinical trials to ensure the success of future vaccine programs.

Nuclear hormones play an important role in the generation of immune responses and pathogen control. As a consequence, when nuclear hormone levels are abnormal, immune responses suffer and risks of infectious diseases (e.g., respiratory syncytial virus (RSV) bronchiolitis) increase [1]. Here we discuss an additional potential consequence; we propose that abnormal nuclear hormone levels in clinical trial study participants may hamper the development of new vaccines.

## 1. The Long Path to RSV Vaccine Licensing

The number of RSV vaccine candidates that have been researched, but that have not achieved licensure, is high [2,3,4]. Pre-clinical tests have been conducted in numerous animal species including mice, cotton rats, lambs, and non-human primates [5]. Whole virus has been tested, following inactivation, cold-adaptation, or other forms of attenuation [6,7,8,9,10,11]. Isolated external and internal proteins have been tested with a variety of adjuvants [2,12,13,14,15,16,17,18]. Protein-based vaccines have most often included the attachment glycoprotein (G) and/or the fusion protein (F), each of which is instrumental in virus–cell interactions. Both secreted and membrane protein structures have been tried. Protein manipulations have included truncation, stabilization, scaffolding, and/or creation of chimeras [19,20,21,22]. Vectors for protein delivery have included Newcastle disease virus, human parainfluenza viruses, Sendai virus, bovine parainfluenza virus-type 3, alphaviruses, adenovirus, vaccinia virus, bacteria, and plants [23,24,25,26,27,28,29,30,31,32,33,34,35,36]. Virus-like particles (VLPs), virosomes, nucleic acid-based vaccines, and peptides have been tried [31,32,33,34,35,37,38,39,40,41,42,43]. Target populations have included infants, older children, expectant mothers, and the elderly. While vaccine success has frequently seemed imminent throughout the decades, no vaccine has reached the finish line despite >50 years of research.

## 2. Vaccine Development Hurdles in Human Immunodeficiency Virus (HIV) and Influenza Virus Fields

RSV vaccines are not the only products for which there have been hurdles in recent years. Human immunodeficiency virus (HIV) vaccine development programs have suffered decades of disappointment with no licensed vaccine product in sight, and previously-licensed vaccines are also in the spotlight. In 2016, the Centers for Disease Control and Prevention (CDC) withdrew its recommendation to administer the FluMist vaccine, a vaccine that had been rigorously promoted in previous years. One study in 2010 showed <10% seroconversion/seroresponse toward H1N1 and H3N2 components among recipients of FluMist [44]. Such results encourage researchers to seek explanations for weak immune responses toward vaccines, with attention not just to vaccine products, but to the immunocompetence of vaccine recipients.

## 3. Will Attention to the Host’s Nuclear Hormone Levels Improve Vaccine Success?

Vaccine developers face numerous challenges, as their products must be proven stable, immunogenic, and safe. Here we consider an additional challenge: insufficient and/or imbalanced nuclear hormones and immunocompetence in vaccine study participants.

The nuclear hormone receptor superfamily comprises two major classes, I and II. Class I receptors are homodimers, exemplified by the estrogen receptor (ERα). Class II receptors are heterodimers, exemplified by the vitamin A and vitamin D receptors (respectively, retinoic acid receptor-retinoid X receptor [RAR-RXR] and vitamin D receptor-retinoid X receptor [VDR-RXR]) [45,46,47,48,49]. Nuclear hormone receptors are best known for their ligand-regulated transcription factor function. They are characterized by an N-terminal domain with activation function (AF-1), a DNA-binding domain (DBD), and a C-terminal ligand-binding domain (LBD) with activation function (AF-2) [50,51,52]. It has often been assumed that nuclear hormone receptors are activated or repressed only by binding their nuclear hormone ligands, but several other mechanisms can dictate a receptor’s activation status [53,54,55,56].

DNA consensus motifs (e.g., retinoic acid response elements [RARE], estrogen response elements [ERE], and androgen response elements [ARE]) define sites for nuclear hormone receptor binding throughout the genome. For example, vitamin A and vitamin D receptors, which share the RXR protein component, often bind a pair of hexameric half-sites, RG(G/T)TCA [57]. The hexamers are usually separated by a spacer, and each receptor has a preferred spacer size. The estrogen receptor binds a consensus palindromic motif GGTCAnnnTGACC, as does the androgen receptor (AGAACAnnnTGTTCT [58,59]). However, rules are not absolute. The binding of receptors to their ligands and to DNA is promiscuous and receptors need not bind DNA directly. Rather, they can be tethered to DNA by other factors [50,60,61]. Although nuclear hormone receptors are best recognized for their transcription factor function, they also confer signals at the cell membrane and by binding a variety of escorts within extra-nuclear compartments [62].

Some nuclear hormone receptors have attracted increased attention in recent years, in part because of dietary and other lifestyle changes that have rendered children and adults deficient or insufficient in vitamins A and D [63]. Whereas vitamin insufficiencies and deficiencies were once considered problems only of the developing world, researchers in developed countries are now discovering frequent cases [64]. Nuclear hormone levels in the human population are not stagnant. Some individuals have hypervitaminosis while others are vitamin deficient. An expectant mother may have an estrogen level that is >100× that of a child. There are also concerns about vitamin transport and function. For example, in the context of obesity, even though serum vitamin A levels may appear to be adequate, there can be dysfunctional trafficking and storage of vitamins in parenchymal tissues, including the lungs [65].

As will be described in more detail below, nuclear hormones have profound influences on adaptive immunity toward pathogens and vaccines. Therefore, we ask whether failed vaccine clinical trials may in some cases be due to weaknesses in the host’s nuclear hormone levels and immunocompetence rather than weaknesses in the vaccine product. Generally, when a vaccine candidate is tested, researchers set a target magnitude and/or target frequency of antibody responses toward the vaccine. Individuals are then recruited into the study and randomized into test and control groups. Study participants may be excluded from vaccination if they have a known treatment (e.g., steroid treatment) or disease that causes immunodeficiency [37]. However, additional aspects of immunocompetence are rarely evaluated. If there are not significant differences between test and control groups or if a target magnitude/frequency of positive antibody responses in the test group is not met, the vaccine may be considered unacceptable for further study. Either the vaccine concept is rejected or vaccine modifications are made to improve the product. Rarely is attention given to the nuclear hormone levels that may render study participants in the test group poorly responsive to vaccines, and rarely is there a positive control of immunocompetence among study participants.

Given that nuclear hormone levels can fluctuate within populations and can influence immunocompetence, it is possible that attractive vaccine candidates are in some cases mistakenly rejected due to weaknesses in study populations. We encourage the future measurement of the hosts’ nuclear hormone levels by vaccine developers to support accurate interpretations of study results and better solutions when immune responses fail. A correction of host weaknesses, not simply product weaknesses, may be necessary to expedite vaccine success.

## 4. Vitamins and the Immune Response

Vitamin levels influence immune responsiveness to vaccines, and abilities to ward off infectious diseases. In one study by Jones et al., vitamin A levels in humans in the United States correlated with the magnitude of IgA antibodies and neutralizing antibodies toward influenza virus [63]. In a separate study, low vitamin levels were correlated with serious disease when children were hospitalized with RSV or human metapneumovirus [66].

When small animals with vitamin A or A+D deficiencies were tested for responses to influenza and parainfluenza virus vaccines, both B cell and T cell responses were significantly reduced compared to controls [67,68,69,70,71,72]. The IgA response in the respiratory tract, a beneficial first-line-of-defense against respiratory and intestinal pathogens, was weakened in vitamin-deficient mice [73,74]. Antibody responses to the Prevnar-13 vaccine (a conjugate vaccine developed against 13 strains of *Streptococcus pneumoniae*) were also poor [75].

The World Health Organization (WHO) acknowledges the health risks associated with vitamin insufficiencies/deficiencies, and therefore supports vitamin supplementation at the time of vaccination in developing countries (although the positive influence of high-dose vitamin supplements on immune responsiveness remains a topic of considerable debate) [76,77,78]. In developed countries, vitamin deficiencies and insufficiencies are perhaps more prevalent than realized [63], and programs focused on correcting deficiencies/insufficiencies in developed countries are limited.

## 5. Sex Hormones and the Immune Response

Outcomes of infectious diseases and vaccination are not the same between the sexes [79,80,81,82,83,84]. A well-publicized example of sex differences is ‘man-flu’, the finding that males suffer more than females from diseases caused by influenza virus [85]. The effect of sex on disease is multi-faceted and may be due to differences in physical barriers as well as the immune response. Our own analyses of influenza virus-infected C57BL/6 mice demonstrated a better virus-specific antibody response in females compared to males, particularly involving the IgG2b subclass [86]. Females also exhibited higher serum antibody levels than males, again involving IgG2b. But the female immune advantage was not absolute; when C567BL/6 mice were vaccinated with the pneumococcus vaccine, Prevnar-13, the males generated the greater magnitude of vaccine-specific antibodies. Such differential effects between sexes and between antigens may be dependent on the site of immunization (intranasal versus intramuscular) and the populations of B cells that are targeted. Sex influences on the immune response exhibit a further level of complexity when nuclear hormone cross-talk is considered. For example, whereas female C57BL/6 mice had greater total serum IgG2b levels compared to males, the preferences were reversed when animals were rendered vitamin A deficient [86].

Altogether, results show that nuclear hormones do not act in isolation. Results encourage comprehensive analyses of nuclear hormones when immune responses are being assessed.

## 6. Innate Immune Cells Are Influenced by Nuclear Hormones

Virtually every mammalian cell is affected by nuclear hormones. It has often been assumed that the adaptive immune response is influenced only indirectly, due to innate immune activities that are affected by hormones. It is well known, for example, that innate lymphoid cells are affected by vitamin A throughout development [87]. Macrophages and epithelial cells each respond to vitamin A, albeit with different outcomes [88]. Vitamin levels influence the expression of CD103 on dendritic cells (DC, as well as on adaptive immune cells), and can, therefore, instruct patterns of DC trafficking and residence [67,70,89]. Each of the innate cell populations, when affected by vitamins, may subsequently influence B cell and T cell functions.

## 7. B Cells Are Influenced by Nuclear Hormones Directly

Might nuclear hormones affect adaptive immunity directly? One effect of estrogen on B cells is the upregulation of activation-induced deaminase (AID), an enzyme required for class switch recombination (CSR) and somatic mutation. This phenomenon has been observed more than once, although researchers disagree on the mechanism of action [90,91].

To further investigate direct influences of vitamin A on B cells, we examined the immunoglobulin heavy chain gene sequence and queried the presence of nuclear hormone response elements within the locus. A first discovery was that Sμ, a site essential for the switching of antibody isotypes from IgM to IgG, IgE, or IgA, defined a hotspot for nuclear hormone response elements [92,93]. We then performed chromatin immunoprecipitation (ChIP) assays using purified murine B cells stimulated for one day, and found peaks of estrogen receptor (ERα) binding activity in Sμ and in regulatory elements including Eμ, and both HS1,2 and HS4 of the 3′ regulatory region, 3′RR [86,93]. These regulatory regions are well known for promotion and modulation of CSR and heavy chain gene expression [94]. There was also binding of ERα in loci for antibody light chains and T cell receptors [86].

**Figure 1:**
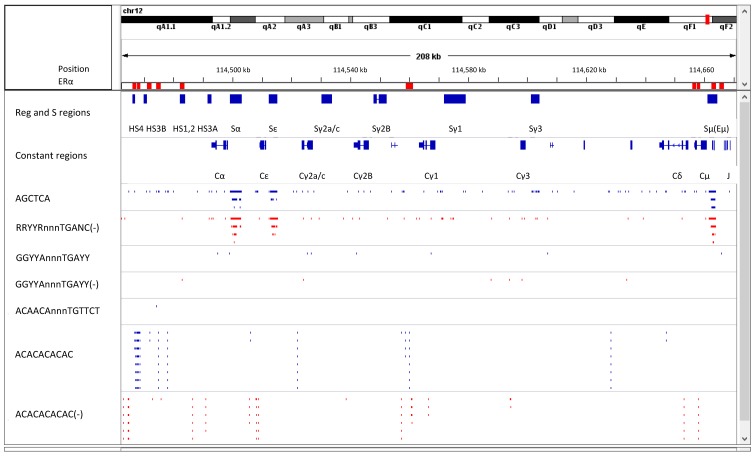
Potential retinoic acid response elements (RARE), estrogen response elements (ERE), and androgen response elements (ARE), ERα binding locations, and CA-rich sequences in the immunoglobulin heavy chain locus. A map of the immunoglobulin heavy chain locus is shown using IGV software. Regulatory regions, S regions, and constant regions are indicated. Note that Eμ is located just upstream of Sμ. The Findseq function was used to identify positions of select sequences. A negative sign indicates the reverse complement. Sequences included AGCTCA (a potential RARE, note one mismatch with the consensus), RRYYRnnnTGANC (a potential ERE), GGYYAnnnTGAYY (a more stringent, potential ERE), ACAACAnnnTGTTCT (a potential ARE, note one mismatch with the consensus), and ACACACACAC (CA-rich regions). Red rectangles indicate positions of major and minor ERα binding peaks, previously identified by ChIP analyses with purified B cells (from C57BL/6 female mice) after a one-day stimulation [86,93]. Not shown are locations for sequences GGACAnnnTGACC upstream of Sγ2b and TGTTAnnnTGACC near HS4.

Figure 1 provides an illustration of these findings. Here, a portion of the immunoglobulin heavy chain locus is mapped using Integrative Genomics Viewer software (IGV, mouse mm9). The reading frame for immunoglobulin genes is oriented from right to left. Switch sites, constant region genes, and components of the 3′RR are shown. Switch sites, by definition, mark positions that are cut and re-ligated after DNA looping to juxtapose V-D-J sequences with *Cγ*, *Cε*, or *Cα* genes (permitting CSR and the respective expression of IgG, IgE, or IgA).

As shown, potential RARE half-sites (AGCTCA, note one mismatch with the consensus sequence described above) and potential EREs (RRYYRnnnTGANC) are prevalent in Sμ, Sε, and Sα regions. Locations of key major and minor peaks of ERα binding within the immunoglobulin heavy chain locus, previously discovered by experiments with one-day stimulated, purified murine B cells, are indicated by red rectangles in Figure 1 [86]. A more stringent, potential ERE (GGYYAnnnTGAYY) coincides with experimentally-proven peaks of ERα binding in HS1,2 and Eμ. The sequence TGTTAnnnTGACC (note two mismatches with the consensus ERE) coincides with the peak of ERα binding to HS4. In addition, a potential ARE (ACAACAnnnTGTTCT) is present in the 3′RR at a site bound by ERα between HS1,2 and HS3B (Figure 1, note one mismatch with the consensus sequence described above).

Figure 1 also shows that repetitive ACACACACAC sequences span the immunoglobulin heavy chain locus and are often adjacent to regions of ERα binding. These CA-rich sequences are reminiscent of the heptamer/nonamer sequences that flank V, D, and J sequences of the antibody locus (e.g., heptamer CACAGTG), required for hairpin formation and juxtaposition of V, D, and J segments during B cell development. We propose that the CA-rich sequences identified in Figure 1 similarly assist DNA looping and gene segment juxtaposition, but in this case for CSR support. When nuclear hormones and other components of enhanceosomes and switchosomes are appropriately engaged (e.g., the Pax 5 and RNA pol II proteins known to associate with the HS1,2 enhancer [86,95,96]), CA-rich DNA interactions may facilitate DNA looping to direct CSR toward a particular S region. We further note that a small peak of ERα binding appears in a central location (approximate position 114,560 kb in Figure 1) upstream of the Sγ2b site and near CA-rich DNA. A potential ERE (GGACAnnnTGACC, note one mismatch with the consensus) coincides with this ERα binding peak. Perhaps ERα binding to this intermediate anchor facilitates CSR at Sγ2b under conditions of high estrogen load, a possible explanation for the IgG2b preference in females. A complex interplay between nuclear hormone ligands, receptors, and response elements, in conjunction with other enhancosome/switchosome members and CA-rich DNA may determine the outcomes of CSR and sex-biased antibody expression patterns.

## 8. Estrogen Response Elements (ERE) and Adjacent Sequences Regulate Antibody Isotype Expression in B Cells

**Figure 2:**
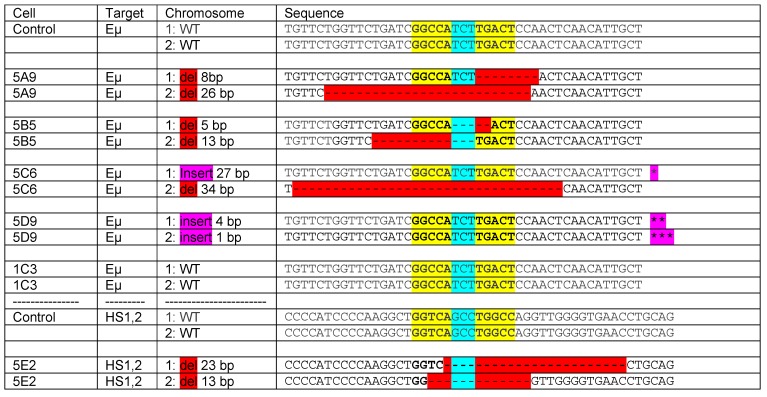
ERE variant sequences among CH12F3 clonal derivatives. CRISPR-Cas9 was used to produce CH12F3 clones with variant ERE. Sequences are shown either in the position of Eμ or HS1,2 for each chromosome from each clone. Red indicates a deletion (del). Purple indicates an insertion (insert). The EREs are shown in yellow with the spacer highlighted in blue. A wild-type CH12F3.5B1 cell subclone served as a control. 1C3 was an additional subclone for which there was no sequence change. Methods: The CH12F3 cell line was obtained from A. Basu and then subcloned by limiting dilution. Cells were grown in Roswell Park Memorial Institute (RPMI) 1640 medium with 10% fetal calf serum (FCS), 2mM glutamine, 2-mercaptoethanol (55 nM) and penicillin/streptomycin (50 units/mL each). The subclone CH12F3.5B1 was selected for further use based on its low surface IgA expression (defined by flow cytometry). ERE variants were introduced into CH12F3.5B1 cells using CRISPR-Cas9 technology. Guide sequences targeting HS1,2 or Eµ were selected and subcloned into px458 or px458-mCherry plasmids as described previously [98,99,100]. Guides were Eµ_Guide_01 (5′-ATGTTGAGTTGGAGTCAAGA-3′) and HS1.2_Guide_01 (5′-CAAGGCTGGTCAGCCTGGCC-3′), each with no potential off-target sites with less than two mismatches. Guide sequences were subcloned into PX458 or PX458-mCherry to generate PX458 _Eµ Guide 01 and PX458-mCherry-HS1.2_Guide 01 plasmids as described previously [100]. These plasmids were then introduced into CHF12F3.5B1 cells using Nucleofector technology (nucleofector program D-023 per manufacturer’s recommendations, Lonza, Basel, Switzerland). Typically, off-target loci with two or more mismatches are not cleaved using this strategy. Cells were cloned by limiting dilution and screened to define the integration event or knockout by targeted next generation sequencing (NGS) using primers SM132.F-5′-tgtgcagagttggctcacaagggca-3′ and SM132.R-5′-ccttgcccatctcctgtcatgtcct-3′ (for the HS1,2 region) or SM133.mIgha.DS.F- taaccgaggaatgggagtga and SM133.mIgha.DS.R-tggactttcggtttggtggg (for the Eµ region) with appropriate Illumina sequencing adaptors. Paired-end 150 bp × 150 bp reads were obtained using Illumina Miseq, joined, and analyzed (Illumina, San Diego, CA, USA). * 27 bp insert ‘CAACCTGGTTGAGACTCCAACTGGTTC’ following the TCT spacer. ** 4 bp insert ‘AATG’ between TC and T of spacer. *** 1 bp insert ‘T’ immediately following the TCT spacer.

**Figure 3:**
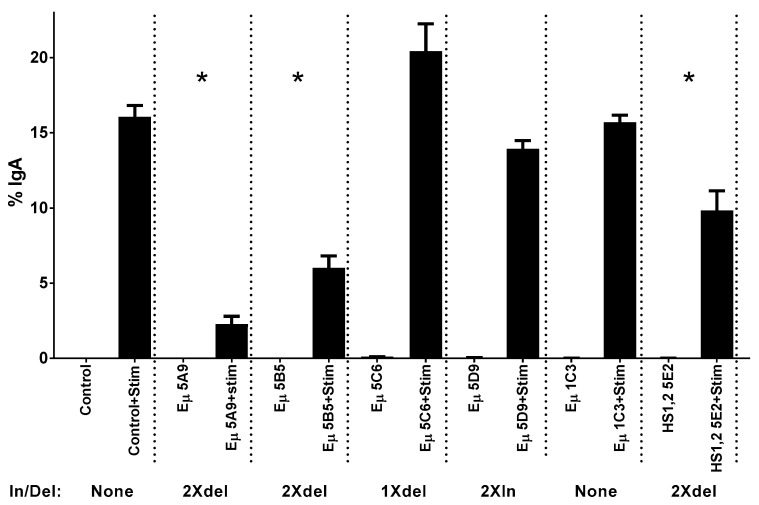
Double deletions of ERE in Eμ or HS1,2 significantly reduce CSR to IgA. Cells were stimulated for 3 days using duplicate wells for each variable. Flow cytometry was performed to examine the frequency of cells expressing membrane IgA before and after stimulation. Percentages of live cells bearing membrane IgA are shown, with means and standard deviations. Controls were CH12F3.5B1 and 1C3, a clonal derivative of CH12F3.5B1 with no ERE sequence change. Insertions (In) or deletions (del) for each cell line are noted (See Figure 2 for sequence details). Unpaired T tests identified significant differences in the percentages of cells bearing membrane IgA after stimulation, when clones with double ERE deletions were compared to the CH12F3.5B1 control (*, *p* < 0.05). Repeat experiments yielded similar results. Methods. Cells were plated in 24 well plates at 5 × 10^4^ cells/well in RPMI with 10% FCS, 2mM glutamine, 2-mercaptoethanol (55 nM) and penicillin/streptomycin (50 units/mL each) for 3 days, with or without a cocktail of recombinant mouse IL-4 (10 ng/mL, Invitrogen, Waltham, MA, USA), anti-CD40 (1 μg/mL R&D Systems, Minneapolis, MN, USA), and TGFβ (2 ng/mL, R&D Systems). For analyses by flow cytometry, cells were pelleted in 1% FCS in phosphate buffered saline [PBS]. Cells were incubated with Fc block (anti-CD16/CD32, BD Biosciences, San Jose, CA, USA) for 20′ on ice. Cells were then pelleted and resuspended in an antibody cocktail including APC conjugated anti-IgM (Invitrogen) and PE-conjugated anti-IgA (eBioscience, San Diego, CA, USA). Incubation was for 30′ on ice. Cells were washed with 1% FCS in PBS and suspended in buffer with 7AAD (Invitrogen) to allow for live/dead cell discrimination. Cells were analyzed on a LSR Fortessa X-20 (BD Biosciences, San Jose, CA, USA). Forward scatter, side scatter, and exclusion of 7AAD were used as parameters to identify live cell populations. Data were evaluated using FCS Express software (6.06.0014, De Novo Software, Glendale, CA, USA).

To test the concept that ERE within regulatory elements influence CSR and antibody isotype expression patterns, we produced ERE variants in CH12F3 B cells (kindly provided by A. Basu) [97]. This cell line undergoes CSR and a switch from IgM to IgA upon stimulation with IL-4, anti-CD40 and TGF-β. ERE variants were introduced into a subclone of CH12F3 (CH12F3.5B1) using clustered regularly interspaced short palindromic repeats (CRISPR) and CRISPR-associated protein-9 nuclease (CRISPR-Cas9) technology. Resultant sequences are shown in Figure 2. Both deletions and insertions were introduced into the ERE, either in Eμ or HS1,2. As shown, two clones had double deletions within the Eμ ERE and 1 clone had a double deletion in the ERE in HS1,2. Other clones maintained at least one chromosome with the wildtype sequence or at least one chromosome with an insertion rather than a deletion. As shown in Figure 3, when clones were stimulated for three days and then assessed for IgA production, the three clones with double deletions within ERE (either in Eμ or HS1,2) were significantly reduced in their ability to produce IgA. In contrast, cells with at least one wildtype ERE or at least one insertion exhibited IgA production comparable to the control. Results show that EREs are critical components of regulatory regions in B cells, and that these sequences dictate antibody isotype expression upon B cell activation. Additional research is encouraged to learn precisely how changes in nuclear hormone levels alter ligand-receptor interactions at these sites. Given the importance of these sites, it makes sense that the binding of ERα to ERE (in conjunction with other nuclear hormones and enhanceosome proteins [95]) will influence the site’s effect on CSR, and that complex cross-talk between nuclear hormones will influence immune responses toward pathogens and vaccines.

## 9. Additional Nuclear Hormones

While we have focused here on vitamins and sex hormones, additional nuclear hormones will influence responses to vaccines and infectious pathogens. Prednisolone, a drug well known for its inhibition of inflammatory responses caused by autoimmune disease, infection, or other forms of tissue injury [101,102] binds a class I glucocorticoid receptor. The thyroid hormones bind class II receptors that share one protein component (RXR) with vitamin A and vitamin D receptors, and modulate both innate and adaptive immune activities [103]. Bidirectional communications between endocrine and immune systems influence a variety of cells including monocytes, macrophages, and lymphocytes [104]. Class III and IV receptors have also been described [56]. The composite of these and other factors will contribute to the cross-talk described above, both within the nucleus and in extra-nuclear compartments, to define immune responses toward pathogens and vaccines.

## 10. Conclusions

We have described the complex influences of nuclear hormones on the immune response and complex interactions between nuclear hormones and the immunoglobulin heavy chain locus. Nuclear hormone receptors bind elements throughout the mammalian genome, dictating a vast array of gene functions. Nuclear hormones will affect the development, maturation, and trafficking of B cells, T cells, and cells of the innate immune system [87,89,105]. Apart from the CSR described here, it is likely that nuclear hormones also influence V-J/V-D-J joining, somatic mutation, and affinity maturation [106,107,108]. With these concepts in mind, we recommend that attention be paid to nuclear hormone levels among vaccine study participants. Possibly, poor immunogenicity of candidate vaccines is sometimes due to insufficiencies of the host, not just the vaccine product. By monitoring nuclear hormone levels among study participants and including positive controls in clinical trials, we may better understand which host populations are capable or incapable of healthy immune responses toward vaccines. A focus on accurate analyses of study data and corrections of insufficiencies that exist in host populations may then expedite the success of vaccine programs.

## Abbreviations

RSV	respiratory syncytial virus
HIV	human immunodeficiency virus
RAR	retinoic acid receptor
RARE	retinoic acid response element
VDR	vitamin D receptor
ERα	estrogen receptor α
ERE	estrogen response element
ARE	androgen response element
AID	activation induced deaminase
CSR	class switch recombination
3′RR	3′ regulatory region
ChIP	chromatin immunoprecipitation
DBD	DNA-binding domain
LBD	ligand-binding domain
FCS	fetal calf serum
NGS	next-generation sequencing
